# Supplementary material for: A Comprehensive Assessment of Ultraviolet-Radiation-Induced Mutations in Flammulina filiformis Using Whole-Genome Resequencing
Source: J Fungi (Basel). 2024 Mar 20;10(3):228. doi: 10.3390/jof10030228 (PMC10971301; doi:10.3390/jof10030228)
Supplement: Supplementary file 1 [file jof-10-00228-s001.zip › Supplementary Material S8/KEGG annotation/out/64381550635650.os/KO/out_map/map05206.html]

KEGG PATHWAY: MicroRNAs in cancer - Reference pathway


|  |  |
| --- | --- |
| **MicroRNAs in cancer - Reference pathway** |  |

[
Pathway menu
| Organism menu
| Pathway entry
| Show description
| User data mapping
]

MicroRNA (miRNA) is a cluster of small non-encoding RNA molecules of 21 - 23 nucleotides in length, which controls gene expression post-transcriptionally either via the degradation of target mRNAs or the inhibition of protein translation. Using high-throughput profiling, dysregulation of miRNAs has been widely observed in different stages of cancer. The upregulation (overexpression) of specific miRNAs could lead to the repression of tumor suppressor gene expression, and conversely the downregulation of specific miRNAs could result in an increase of oncogene expression; both these situations induce subsequent malignant effects on cell proliferation, differentiation, and apoptosis that lead to tumor growth and progress. The miRNA signatures of cancer observed in various studies differ significantly. These inconsistencies occur due to the differences in the study populations and methodologies used. This pathway map shows the summarized results from various studies in 9 cancers, each of which is presented in a review article.

|  |  |
| --- | --- |
| Reference pathway | 100% |
